# What Do Randomised Trials Reveal About Robotic Surgery? A Critical Appraisal Across Colorectal, Upper Gastrointestinal, Hepato-Pancreaticobiliary, and General Surgical Specialties

**DOI:** 10.3390/jcm14196699

**Published:** 2025-09-23

**Authors:** Georgios Geropoulos, Samuel Massias, Qamil Pajaziti, Bibechan Thapa, Syed Nouman Mohsin, Vanash Patel

**Affiliations:** 1 Department of Surgery, West Hertfordshire Teaching Hospitals NHS Trust, Watford General Hospital, Watford WD18 0HB, UK; 2 Department of Surgery and Cancer, Imperial College London, St Mary’s Hospital, London W2 1NY, UK

**Keywords:** cost-effectiveness, robotic-assisted surgery, surgical outcome

## Abstract

Robotic-assisted surgery has become an established modality across various surgical specialties, offering enhanced visualisation, precision, and ergonomics compared to conventional laparoscopic approaches. This review summarises current evidence from randomised controlled trials evaluating robotic surgery in colorectal, upper gastrointestinal, hepatopancreatobiliary, hernia, bariatric, and breast procedures. The findings consistently show that robotic surgery is safe and technically feasible, with comparable or improved short-term outcomes in select contexts, particularly in anatomically complex cases such as rectal cancer or pancreatic surgery. However, most trials report significantly longer operative times and higher direct costs, with limited demonstration of clear cost-effectiveness or long-term oncological superiority. Consequently, while robotic surgery holds promise for selected indications, its widespread adoption should be guided by ongoing evidence generation and careful consideration of clinical value, patient outcomes, and economic impact.

## 1. Introduction

Robotic-assisted surgery has evolved from a novel innovation into a mainstream surgical modality over the past two decades. Robotic-assisted surgery has evolved over the past three decades, beginning with early systems such as AESOP and ZEUS in the 1990s, which laid the foundation for computer-assisted surgical platforms. However, the introduction and FDA approval of the da Vinci Surgical System in 2000 marked the first widespread clinical adoption of robotic surgery, initially in urological and gynaecological procedures, before its expansion into general surgery [1,2]. These systems offer several key technical advantages over conventional laparoscopic surgery, including enhanced three-dimensional magnified vision, tremor filtration, motion scaling, and wristed instrumentation that allows greater dexterity in confined anatomical spaces [3]. These attributes make robotic surgery particularly appealing in anatomically challenging procedures, such as total mesolectal excision for rectal cancer, or operations requiring fine dissection near critical structures, such as the recurrent laryngeal nerve or the autonomic pelvic plexus [4,5].

Over time, robotic surgery has seen growing adoption across a broad range of surgical specialties, including colorectal, upper gastrointestinal (UGI), hepatopancreatobiliary (HPB), bariatric, and general surgery [6,7]. Its use has been supported by improvements in surgical ergonomics, enhanced visualisation, and the potential for standardisation of complex tasks. However, early adoption was largely based on observational studies and institutional experiences, which demonstrated feasibility and technical safety but lacked robust comparative evidence. In response, an increasing number of randomised controlled trials (RCTs) have been conducted to rigorously evaluate the role of robotic surgery across various indications. The aim of this review is to explore the current evidence from these randomised trials and critically assess the clinical outcomes associated with robotic surgery.

## 2. Materials and Methods

This study was conducted as a narrative review of randomised controlled trials (RCTs) evaluating robotic surgery compared to laparoscopic or open approaches across colorectal, upper gastrointestinal (UGI), hepatopancreatobiliary (HPB), bariatric, breast, and general surgery. A structured literature search was performed in PubMed, EMBASE, and Google Scholar, covering the period from January 2000 to June 2025. Search terms (including MeSH) included: robotic surgery, laparoscopic surgery, open surgery, randomized controlled trial, colorectal, gastrectomy, esophagectomy, pancreatectomy, cholecystectomy, hernia repair, and mastectomy. Reference lists of relevant articles and systematic reviews were also screened to identify additional eligible trials. Table 1 summarises the basic characteristics of the included studies.

Two authors independently screened titles and abstracts, followed by full-text review to determine eligibility. Only peer-reviewed RCTs involving adult patients were included. Non-randomised studies, conference abstracts, and animal studies were excluded. For each trial, data were extracted on study design, sample size, surgical specialty, operative time, perioperative morbidity, oncological adequacy (e.g., resection margins, lymph node yield), length of hospital stay, functional outcomes, and cost-effectiveness. Disagreements were resolved by consensus.

The synthesis process was qualitative and descriptive, focusing on the methodological rigour and reported clinical outcomes of included RCTs. Given the heterogeneity of surgical procedures and outcomes assessed, meta-analysis was not performed. The methodological quality of the included trials was evaluated using the RoB 2 (Risk of Bias 2.0) tool, which assesses potential bias across five domains: randomization process, deviations from intended interventions, missing outcome data, measurement of the outcome, and selection of the reported result. Each domain was judged as low risk, some concerns, or high risk of bias [21].

## 3. Robotic Colorectal Surgery

The ROLARR (Robotic vs. Laparoscopic Resection for Rectal Cancer) trial was a multicentre, international, randomised clinical study involving 471 patients across 29 sites in 10 countries, comparing robotic-assisted and conventional laparoscopic surgery for rectal cancer [4]. The primary outcome, conversion to open laparotomy, occurred in 8.1% of patients in the robotic group versus 12.2% in the laparoscopic group (*p* = 0.16), a difference that was not statistically significant. Operative time was longer for robotic surgery (mean difference: +51 min, *p* = 0.001), and hospital stay was similar between groups (median 7 days). Circumferential resection margin positivity was comparable (5.1% robotic versus 6.3% laparoscopic), as were intraoperative and postoperative complication rates. Thirty-day mortality was low and equal (0.9% in both arms), with four deaths due to septic complications. At six months, bladder and sexual function scores showed no significant difference between groups in both male and female patients. From a health economic perspective, robotic surgery incurred significantly higher costs, with a mean of $13,668 compared with $12,556 for laparoscopy (mean difference: $1132; *p* = 0.02). This was primarily attributed to longer operating room time and higher instrument costs. The study concluded that while robotic surgery is safe and feasible, it did not significantly reduce conversion rates or improve functional outcomes and incurs greater cost, limiting its clinical advantage over conventional laparoscopy in rectal cancer surgery.

The REAL trial was a large multicentre randomised clinical study conducted across 11 centres in China to evaluate long-term outcomes of robotic versus laparoscopic surgery for middle and low rectal cancer [8]. A total of 1240 patients were randomised, and analysis showed that robotic surgery significantly reduced the 3-year locoregional recurrence rate (1.6% versus 4.0%; HR 0.45, *p* = 0.03) and improved disease-free survival (87.2% versus 83.4%; *p* = 0.04), while overall survival was similar. Robotic surgery was also associated with significantly fewer perioperative complications (16.1% versus 22.9%; *p* = 0.003) and reduced intraoperative blood loss and conversion rates. Functional outcomes, including urinary, sexual, and defecation function, were significantly better in the robotic group, particularly in the first postoperative year. This trial provides the first high-quality evidence suggesting that robotic rectal cancer surgery not only enhances short-term recovery but may also offer long-term oncological and functional benefits compared to conventional laparoscopy.

The COLRAR (Comparison of Laparoscopic Versus Robot-Assisted Surgery for Rectal Cancers) RCT evaluated the surgical outcomes of robot-assisted total mesorectal excision (TME) versus laparoscopic TME in 295 patients with mid to low rectal adenocarcinoma located less than 10 cm from the anal verge [9]. The trial found no significant difference in the rate of complete TME between the robotic and laparoscopic groups (80.7% versus 77.1%, respectively). Overall pathological outcomes, including lymph node yield and negative circumferential resection margins, were similar, although a subanalysis revealed a lower rate of positive margins in the robotic group (0% versus 6.1%; *p* = 0.031). Postoperative complication rates were comparable (12.0% versus 8.3%), while opioid use duration was shorter in the robotic arm (*p* = 0.028). Despite early trial termination due to slow accrual, these findings suggest robotic surgery offers certain technical advantages, but does not confer a clear overall improvement in oncological or perioperative outcomes compared to laparoscopy.

Feng et al. from Zhongshan Hospital, Fudan University, conducted a single-centre RCT comparing robotic and laparoscopic abdominoperineal resections (APRs) for low rectal cancer [10]. A total of 347 patients with tumours ≤5 cm from the anal verge were randomised to robotic (*n* = 174) or laparoscopic (*n* = 173) APR. Robotic surgery was associated with a significantly lower 30-day postoperative complication rate (13.2% versus 23.7%; *p* = 0.013), reduced blood loss, fewer conversions to open surgery, shorter hospital stay (median 5 versus 7 days; *p* < 0.001), and improved urinary and sexual function. Long-term oncological outcomes were similar between groups.

The SIRIRALS trial published in 2024, investigated the surgical stress response in patients undergoing robot-assisted versus laparoscopic surgery for colon cancer [22]. The study concluded that the robotic approach was associated with a reduced systemic stress response, as evidenced by lower postoperative C-Reactive Protein (CRP) levels on the first postoperative day.

## 4. Robotic Mastectomy

In a phase III RCT conducted at the European Institute of Oncology in Milan, Italy, 80 women with breast cancer or BRCA mutations were randomised to undergo robotic or open nipple-sparing mastectomy [17]. The study found no significant differences in complication rates between the two groups, confirming the surgical safety of the robotic approach. Although the robotic procedure took significantly longer (by 78 min), patients in the robotic group maintained their preoperative quality of life, with higher scores in breast satisfaction and psychosocial, physical and sexual wellbeing at 12 months. In contrast, patients undergoing open surgery experienced a decline in these domains. Body image and nipple sensitivity were also better preserved in the robotic group. At a median follow-up of 28.6 months, no local recurrences were reported, supporting the early oncological safety of robotic nipple-sparing mastectomy.

## 5. Robotic Hernia Surgery

The PROVE-IT trial compared patient reported outcomes between robotic and laparoscopic ventral hernia repairs with intraperitoneal mesh [23]. The study found no significant differences in postoperative pain, quality of life, or overall satisfaction between the two approaches. However, the robotic approach was associated with longer operative times and higher costs.

The ORREO trial compared robotic retromuscular ventral hernia repair with open retromuscular ventral hernia repair and found no significant differences in postoperative complication rates or patient reported outcomes between the two approaches [18]. Robotic surgery was associated with a shorter hospital stay but required longer operative time and resulted in higher overall costs, although the cost difference was not statistically significant. Both groups demonstrated significant improvements in quality of life and pain scores over time.

A multicentre, blinded RCT by Dhanani et al. compared robotic versus laparoscopic ventral hernia repair in 124 patients, with 101 completing two-year follow-up [19]. The study found no significant differences in surgical site infection, surgical site occurrences, or hernia recurrence (4% in robotic versus 13% in laparoscopic; *p* = 0.124). However, reoperation rates were significantly lower in the robotic group (0% versus 11%; *p* = 0.019), suggesting a potential long-term advantage. Patient-reported outcomes at two years, including functional status, pain, and satisfaction with repair and cosmesis, improved in both groups with no significant differences between them. Although this trial was not powered for recurrence or reoperation, its findings suggest that robotic repair may offer similar or improved long-term outcomes.

Miller et al. conducted the RIVAL trial, a unique and notable multicentre randomised clinical study that specifically compared robotic and laparoscopic transabdominal preperitoneal (TAPP) inguinal hernia repair, making it one of the few trials in this subspecialty to report long-term outcomes [20]. Conducted across six centres in the United States, the study included 102 patients, with follow-up rates of 81% at one year and 75% at two years. The results demonstrated no significant differences between robotic and laparoscopic approaches in terms of postoperative pain, neuropathic pain, quality of life, physical activity, wound morbidity, or hernia recurrence. At 2 years, recurrence rates were equal (one in each group), confirming that both techniques yield equivalent long-term outcomes when performed by experienced surgeons.

## 6. Robotic Bariatric Surgery

A comprehensive systematic review and meta-analysis examined the outcomes of robotic versus laparoscopic bariatric surgery [24]. The analysis included multiple RCTs and observational studies, focusing on perioperative outcomes such as operative time, complication rates, and length of hospital stay. The findings suggested that while robotic bariatric surgery may offer certain technical advantages, it often involves longer operative times and higher costs compared to laparoscopic surgery. However, complication rates and length of hospital stay were comparable between the two approaches.

In summary, while randomised clinical trials specifically focusing on robotic bariatric surgery are limited, existing studies suggest that robotic approaches are feasible and safe, with outcomes comparable to traditional laparoscopic methods. However, considerations such as longer operative times and higher costs remain pertinent. Ongoing research and further RCTs are needed to comprehensively evaluate the benefits and limitations of robotic bariatric surgery.

## 7. Robotic Upper Gastrointestinal Surgery

The REVATE (Robotic Oesophagectomy versus Video-Assisted Thoracoscopic Esophagectomy) trial was a multicentre RCT involving 203 patients with oesophageal squamous cell carcinoma across three high-volume Asian centres [13]. The trial demonstrated that robot-assisted oesophagectomy achieved a significantly higher success rate in left recurrent laryngeal nerve lymph node dissection compared to video-assisted oesophagectomy (88.3% versus 69%, *p* < 0.001) and a lower rate of permanent nerve palsy at six months (5.8% versus 20%, *p* = 0.003). Oncological outcomes were comparable, with high R0 resection rates in both groups (94.2% in robotic versus 98% in video-assisted, *p* = 0.280). Robotic surgery was associated with a greater number of dissected mediastinal lymph nodes (median 16 versus 14, *p* = 0.035), though total lymph node yield did not differ significantly. Anastomotic leak rates were higher in the robotic group (11.7% versus 6%), but the difference was not statistically significant. Postoperative recovery, complication rates, and pneumonia incidence were similar between groups. The trial supports technical advantages of robotic surgery in nodal dissection and nerve preservation, though its impact on long-term outcomes and cost-effectiveness remains to be determined.

This Brazilian single-centre RCT compared robotic gastrectomy (RG) versus open gastrectomy (OG) with D2 lymphadenectomy in 60 patients with stage cT2–4N0–1 gastric adenocarcinoma [14]. The study found that RG was non-inferior to OG regarding short-term surgical outcomes. Both groups achieved 100% R0 resection, and there were no significant differences in the number of harvested lymph nodes, postoperative complications, readmissions, or hospital stay. However, RG was associated with significantly reduced intraoperative bleeding (*p* < 0.001) and longer operative time (*p* < 0.001). These findings support the safety and feasibility of robotic surgery for gastric cancer in Western settings, though long-term oncological outcomes are still pending.

## 8. Robotic Hepatopancreatobiliary Surgery

This multicentre RCT, conducted across three high-volume hospitals in China, compared robotic pancreaticoduodenectomy (RPD) with open pancreaticoduodenectomy (OPD) in 161 patients with resectable benign, premalignant, or malignant tumours of the pancreatic head or periampullary region [15]. The study demonstrated that RPD was associated with a significantly shorter postoperative hospital stay compared to OPD (median 11.0 versus 13.5 days; *p* = 0.029). No significant differences were observed between the groups in 90-day mortality (1% in each group), incidence of severe postoperative complications (Clavien–Dindo grade ≥3), or readmission rates (7% RPD versus 6% OPD). Both surgical approaches exhibited comparable safety profiles when performed by experienced surgeons. While short-term outcomes favoured RPD in terms of recovery, the study did not report oncological results or long-term follow-up, and further research is required to evaluate survival and recurrence outcomes.

The first RCT comparing robotic versus open simultaneous resection of rectal cancer and synchronous liver metastases enrolled 171 patients (86 robotic, 85 open) at a single high-volume centre in China [16]. The trial demonstrated that robotic surgery resulted in significantly fewer 30-day postoperative complications (31.4% versus 57.6%, *p* = 0.014), including a lower incidence in major complications (8.1% versus 20%, *p* = 0.029), with no mortality in either group. Robotic surgery was associated with reduced blood loss, faster recovery of bowel function, shorter hospital stay (mean 8.0 versus 10.7 days, *p* < 0.001), and better preservation of bladder and sexual function at three months. Oncological outcomes were comparable, with 100% R0 resection rates for both rectal and liver lesions, similar lymph node yields, and no significant differences in three-year disease-free survival (39.5% versus 35.3%) or overall survival (76.7% versus 72.9%). Although total hospital costs were higher for robotic surgery, postoperative costs were lower, and recovery was enhanced. These findings support the use of robotic surgery as a safe and effective alternative for selected patients with synchronous colorectal liver metastases, offering superior short-term outcomes without compromising oncological results.

## 9. Endorsement Status of Robotic Surgery in Major Colorectal Guidelines

The latest Enhanced Recovery After Surgery (ERAS) Society Recommendations for colorectal surgery published in 2018 support evidence for robotic colorectal surgery [25]. Robotic surgery has not been shown to improve survival compared with laparoscopic surgery, and long-term survival data remain lacking. For colonic resections, robotic surgery offers no significant advantages over standard laparoscopy, while incurring increased costs. In rectal resection, while robotic surgery has been evaluated through meta-analysis and randomised trials, it showed no significant differences in outcomes aside from a lower conversion rate and was found to be more expensive and not cost-effective.

For instance, the European Society of Coloproctology highlighted the ROLARR trial as the largest randomised study comparing robotic and laparoscopic approaches for curative rectal cancer surgery. Subsequent guidelines, such as the 2020 Clinical Practice Guidelines for the Management of Rectal Cancer by the American Society of Colon and Rectal Surgeons, have incorporated evidence from studies like ROLARR to inform their recommendations. These guidelines typically assess the efficacy and safety of different surgical approaches, including robotic-assisted and laparoscopic techniques, to provide evidence-based recommendations for clinical practice.

Despite the increasing adoption of robotic surgery for colorectal cancer and the emergence of high-quality RCTs—such as the REAL, COLRAR, and ROLARR trials—major international guidelines, including those from the National Comprehensive Cancer Network (NCCN), European Society for Medical Oncology (ESMO), and Japanese Society for Cancer of the Colon and Rectum (JSCCR), have not yet incorporated specific recommendations based on these studies. Current guidelines continue to support laparoscopic total mesorectal excision as an acceptable standard but stop short of endorsing robotic-assisted approaches, primarily due to limited long-term evidence and cost-effectiveness concerns. Although the REAL trial demonstrated improved locoregional control and functional outcomes with robotic surgery, formal guideline revisions remain pending further validation and broader international consensus.

## 10. Limitations

The majority of RCTs included in this review adhered to high methodological standards, incorporating appropriate randomisation methods, clearly defined eligibility criteria, and intention-to-treat analyses. Trials such as ROLARR, REAL, and PROVE-IT were multicentre in design, with clear reporting of primary and secondary outcomes, sample size calculations, and follow-up protocols, supporting their internal validity. Blinding was generally not feasible due to the nature of surgical interventions, but outcome assessment was often performed by independent parties or through objective measures (e.g., pathological margins, complication rates), minimising detection bias. Reporting quality was generally robust, with most trials adhering to CONSORT guidelines and registering their protocols prospectively in trial registries such as ClinicalTrials.gov.

Nevertheless, some limitations in methodological quality were observed across studies. Several trials, particularly those conducted in single-centre settings or involving highly selected populations (e.g., robotic mastectomy or bariatric surgery), were underpowered or lacked blinding of outcome assessors. Early termination due to slow recruitment, as in the COLRAR trial, also limited both the generalizability and the statistical power of the findings. Furthermore, few trials included long-term oncological follow-up or comprehensive cost-effectiveness analyses, which are essential for informing health policy. Overall, while the evidence base for robotic surgery is expanding and includes several high-quality RCTs, further large-scale, blinded, and long-term studies are required to confirm both clinical benefit and economic justification across surgical subspecialties.

In support of these observations, a structured risk of bias assessment was conducted using an adapted version of the ROBINS-I tool, applied to evaluate key methodological domains in the included randomised trials. Overall, the methodological quality of the trials was considered to be high. Most studies demonstrated a low risk of bias across critical domains, including confounding, participant selection, classification of interventions, and outcome measurement. Notably, large multicentre trials such as ROLARR, REAL, and RIVAL exhibited robust design features and consistently low risk across all assessed categories. Minor concerns were noted in a small number of studies, primarily in the domain of missing data, particularly among single-centre trials or those with incomplete follow-up reporting (e.g., Park et al., Miller et al.). Nonetheless, these limitations were isolated and did not appear to systematically compromise the internal validity of the findings. Full results of the quality assessment are presented in Table 2.

## 11. Conclusions

Robotic-assisted surgery has emerged as a technically feasible and increasingly adopted modality across general, colorectal, upper gastrointestinal, hepatopancreatobiliary, and hernia procedures. The current body of RCT evidence reveals that robotic surgery consistently demonstrates comparable safety profiles to conventional laparoscopic or open approaches, with similar or slightly reduced rates of perioperative complications in selected contexts, particularly in rectal and pancreatic surgery. Notably, trials such as REAL and the robotic APR study from Zhongshan Hospital reported significantly lower postoperative morbidity and enhanced functional recovery, highlighting potential advantages in carefully selected patients and procedures involving confined pelvic anatomy or complex dissection planes.

However, robotic procedures are universally associated with longer operative times, with several trials (e.g., ROLARR, COLRAR, and robotic gastrectomy) reporting increased durations often exceeding 30–60 min compared to conventional approaches. From an oncological perspective, robotic surgery offers at least equivalent outcomes in terms of circumferential resection margin status, lymph node yield, and R0 resection rates. The REAL trial provides the strongest oncological evidence to date, showing a statistically significant improvement in locoregional control and disease-free survival in robotic rectal cancer surgery. Nonetheless, these benefits have not been uniformly observed across all tumour types or surgical sites, and long-term survival data remain sparse in most domains.

One of the most persistent and unresolved limitations of robotic surgery is its economic impact. Across nearly all included trials—such as ROLARR, PROVE-IT, and those evaluating right colectomy and hernia repair—robotic procedures incur significantly higher direct costs, largely driven by equipment expenses, longer operative times, and consumable use. Few studies have demonstrated clear cost-effectiveness in terms of either reduced downstream complications or improved quality of life. Thus, while robotic surgery is clinically safe and oncologically sound, the higher financial burden remains a major consideration for health systems and guideline developers.

An important recurring theme across the reviewed trials is the consistently higher cost of robotic surgery compared with laparoscopic approaches. Multiple cost analyses and systematic reviews have shown that robotic procedures are associated with significantly greater operative and hospitalisation expenses, largely driven by longer operating room times, higher equipment costs, and consumables [4,16]. While some studies suggest potential downstream benefits, such as reduced length of stay, lower conversion rates, or improved functional outcomes in select settings (e.g., rectal cancer surgery), these advantages have not been sufficient to offset the higher upfront expenditure in most economic models [1,2,3]. Consequently, the financial sustainability and cost-effectiveness of robotic surgery remain unresolved issues, particularly in resource-limited healthcare systems. Future trials incorporating robust health economic endpoints, including quality-adjusted life years (QALYs) and long-term cost analyses, are essential to determine whether the clinical gains offered by robotics can justify their financial burden across different surgical specialties.

In summary, robotic-assisted surgery holds clear potential to refine surgical care, particularly in anatomically challenging cases. Still, its broader adoption must be balanced against the trade-offs of longer operative times and higher procedural costs. Looking ahead, rectal cancer surgery and complex hepatopancreatobiliary procedures appear to represent the most promising fields for future robotic implementation, where the unique technical advantages of robotic systems—such as improved dexterity and visualisation in confined anatomical spaces—may translate into meaningful improvements in patient outcomes once further validated by large-scale, long-term trials.

## Figures and Tables

**Table 1:** Basic characteristics of the included studies.

First Author (Trial)	Specialty	Sample Size (n)	Intervention	Comparator	Key Findings
JAYNE (ROLARR), 2017 [4]	Colorectal (rectal cancer)	471	Robotic rectal resection	Laparoscopic rectal resection	Conversion rate not significantly lower (8.1% vs. 12.2%); longer operative time (+51 min); similar CRM positivity; higher cost for robotic surgery.
FENG (REAL), 2022 [8]	Colorectal (rectal cancer)	1240	Robotic TME	Laparoscopic TME	Lower 3-year locoregional recurrence (1.6% vs. 4.0%); improved DFS (87.2% vs. 83.4%); fewer perioperative complications; improved functional outcomes.
PARK (COLRAR), 2018 [9]	Colorectal (rectal cancer)	295	Robotic TME	Laparoscopic TME	No difference in complete TME (80.7% vs. 77.1%); lower CRM positivity (0% vs. 6.1%); similar complications; shorter opioid use in robotic group.
FENG, 2022 [10]	Colorectal (APR for low rectal cancer)	347	Robotic APR	Laparoscopic APR	Lower complication rate (13.2% vs. 23.7%); reduced blood loss; shorter stay (5 vs. 7 days); improved urinary and sexual function.
PARK, 2012 [11]	Colorectal (right colectomy)	70	Robotic right colectomy	Laparoscopic colectomy	Similar complications, pain, oncologic outcomes; longer operative time (195 vs. 130 min); higher costs for robotic group.
DOHRN, 2022 [12]	Colorectal (right colectomy, ICA vs. ECA)	89	Robotic ICA	Robotic ECA	No difference in QoR-15 recovery scores or complications; anastomosis construction longer in ICA group.
CHAO (REVATE), 2021 [13]	Upper GI (esophagectomy)	203	Robotic esophagectomy	VATS esophagectomy	Improved nerve preservation; more mediastinal LN harvested; similar oncologic outcomes; higher (not significant) anastomotic leak rate.
RIBEIRO, 2022 [14]	Upper GI (gastrectomy)	60	Robotic D2 gastrectomy	Open D2 gastrectomy	Non-inferior; less blood loss; longer operative time; oncologic outcomes equivalent.
LIU, 2020 [15]	HPB (pancreaticoduodenectomy)	161	Robotic PD	Open PD	Shorter hospital stay (11 vs. 13.5 days); similar 90-day mortality and complications; safety confirmed.
CHANG, 2023 [16]	HPB + colorectal (synchronous rectal + liver metastases)	171	Robotic combined resection	Open combined resection	Lower complication rate (31% vs. 58%); shorter stay (8 vs. 11 days); better functional recovery; similar oncologic outcomes.
TOESCA, 2022 [17]	Breast (nipple-sparing mastectomy)	80	Robotic NSM	Open NSM	Similar complication rates; longer operative time (+78 min); better QoL, body image, and nipple sensitivity preserved in robotic group.
WARREN (ORREO), 2024 [18]	Hernia (ventral hernia, retromuscular)	101	Robotic repair	Open repair	Similar complication rates and PROMs; shorter hospital stay; longer operative time; higher costs.
DHANANI (PROVE-IT), 2023 [19]	Hernia (ventral hernia, IPOM)	124	Robotic repair	Laparoscopic repair	Similar pain, QoL, complications; longer operative time; higher costs.
MILLER (RIVAL), 2023 [20]	Hernia (inguinal TAPP)	102	Robotic TAPP repair	Laparoscopic TAPP repair	No differences in pain, QoL, recurrence; longer operative time; higher cost for robotic surgery.

**Table 2:** Quality assessment of robotic general surgery clinical trials included in this study.

Trial	Bias Arising from Randomization Process	Bias Due to Deviations from Intended Interventions	Bias Due to Missing Outcome Data	Bias in Measurement of the Outcome	Bias in Selection of the Reported Result	Overall Risk of Bias
ROLARR (Jayne et al.) [4]	Low risk	Some concerns	Low risk	Low risk	Low risk	Some concerns
REAL (Feng et al.) [8]	Low risk	Low risk	Low risk	Low risk	Low risk	Low risk
COLRAR (Park et al.) [9]	Low risk	Some concerns (early termination)	Low risk	Low risk	Low risk	Some concerns
Robotic APR (Feng et al.) [10]	Low risk	Low risk	Low risk	Low risk	Low risk	Low risk
Park et al. (2012) [11]	Low risk	Low risk	Low risk	Low risk	Low risk	Low risk
Dohrn et al., 2022 [12]	Low risk	Low risk	Low risk	Low risk	Low risk	Low risk
Robotic Mastectomy (Toesca et al.) [17]	Low risk	Some concerns (blinding not feasible)	Low risk	Low risk	Low risk	Some concerns
PROVE-IT (Dhanani et al.) [19]	Low risk	Some concerns	Low risk	Low risk	Low risk	Some concerns
ORREO (Warren et al.) [18]	Low risk	Some concerns	Low risk	Low risk	Low risk	Some concerns
RIVAL (Miller et al.) [20]	Low risk	Low risk	Low risk	Low risk	Low risk	Low risk
Robotic Gastrectomy (Ribeiro et al.) [14]	Low risk	Low risk	Low risk	Low risk	Low risk	Low risk
REVATE (Chao et al.) [13]	Low risk	Some concerns (performance bias)	Low risk	Low risk	Low risk	Some concerns
Robotic Pancreaticoduodenectomy (Liu et al.) [15]	Low risk	Low risk	Low risk	Low risk	Low risk	Low risk
Simultaneous Rectal + Liver Resection (Chang et al.) [16]	Low risk	Low risk	Low risk	Low risk	Low risk	Low risk

## Data Availability

The datasets used and analysed during the current study are available from the corresponding author upon reasonable request.

## Abbreviations

The following abbreviations are used in this manuscript:

APR	Abdominoperineal Resection
BRCA	Breast Cancer Gene
COLRAR	Comparison of Laparoscopic Versus Robot-Assisted Surgery for Rectal Cancers
CONSORT	Consolidated Standards of Reporting Trials
CRP	C-Reactive Protein
ERAS	Enhanced Recovery After Surgery
ESMO	European Society for Medical Oncology
HPB	Hepatopancreatobiliary
JSCCR	Japanese Society for Cancer of the Colon and Rectum
NCCN	National Comprehensive Cancer Network
OG	Open Gastrectomy
OPD	Open Pancreaticoduodenectomy
RCT	Randomised Controlled Trial
REAL	Robotic versus Laparoscopic Surgery for Rectal Cancer in China
REVATE	Robotic Oesophagectomy versus Video-Assisted Thoracoscopic Esophagectomy
RIVAL	Robotic vs. Laparoscopic Transabdominal Preperitoneal Inguinal Hernia Repair
ROBINS-I	Risk Of Bias In Non-randomised Studies-of Interventions
ROLARR	Robotic vs. Laparoscopic Resection for Rectal Cancer
RPD	Robotic Pancreaticoduodenectomy
TAPP	Transabdominal Preperitoneal
TME	Total Mesorectal Excision
UGI	UGI Upper Gastrointestinal Surgery
